# Supplementary figures and images for: Cryo-electron microscopy structures of the N501Y SARS-CoV-2 spike protein in complex with ACE2 and 2 potent neutralizing antibodies
Source: PLoS Biol. 2021 Apr 29;19(4):e3001237. doi: 10.1371/journal.pbio.3001237 (PMC8112707; doi:10.1371/journal.pbio.3001237)

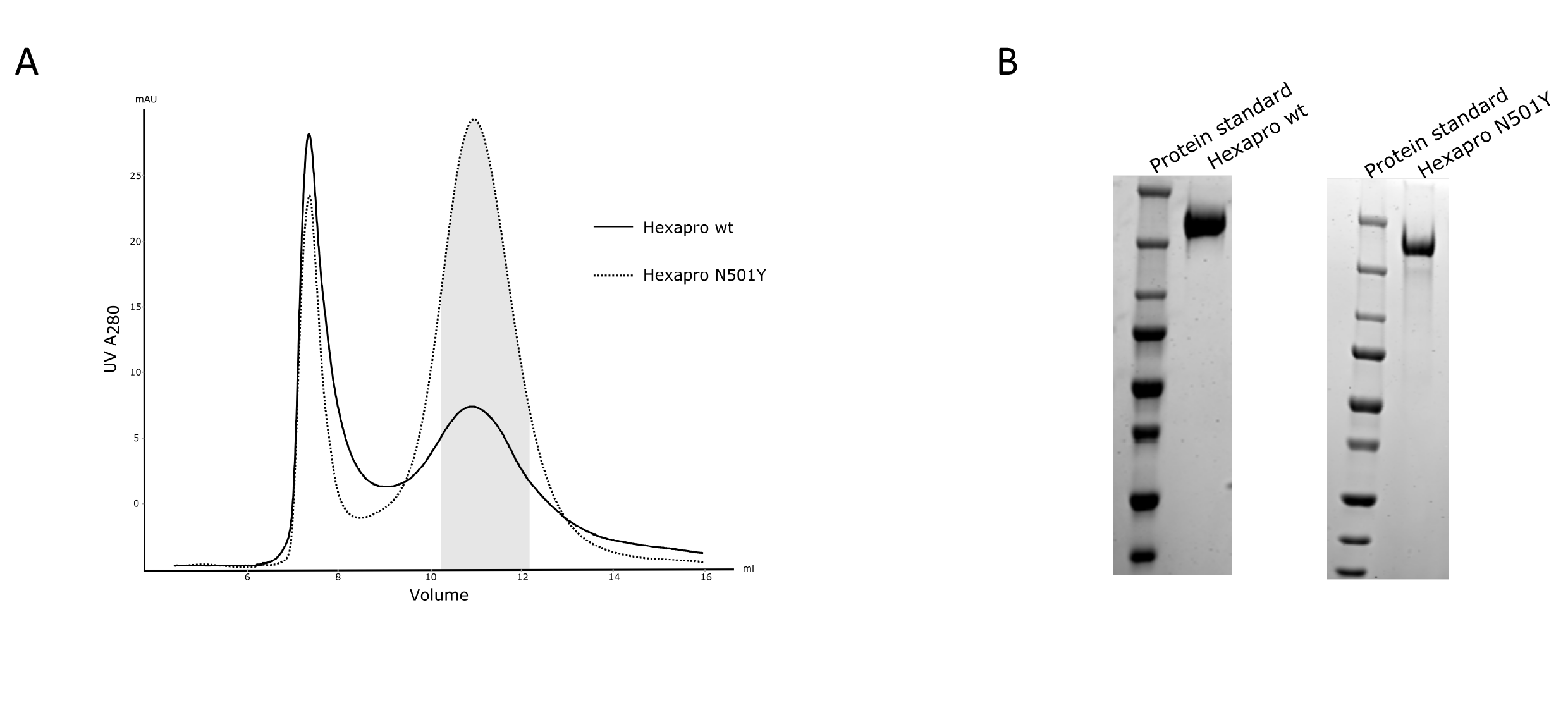

Supplement: S1 Fig — (A) Size exclusion chromatography profile of the indicated affinity-purified HexaPro constructs. Fractions pooled for structural and biochemical studies are indicated by gray shading. (B) SDS-PAGE analysis of pooled and concentrated HexaPro constructs. (TIF) [file pbio.3001237.s001.tif]

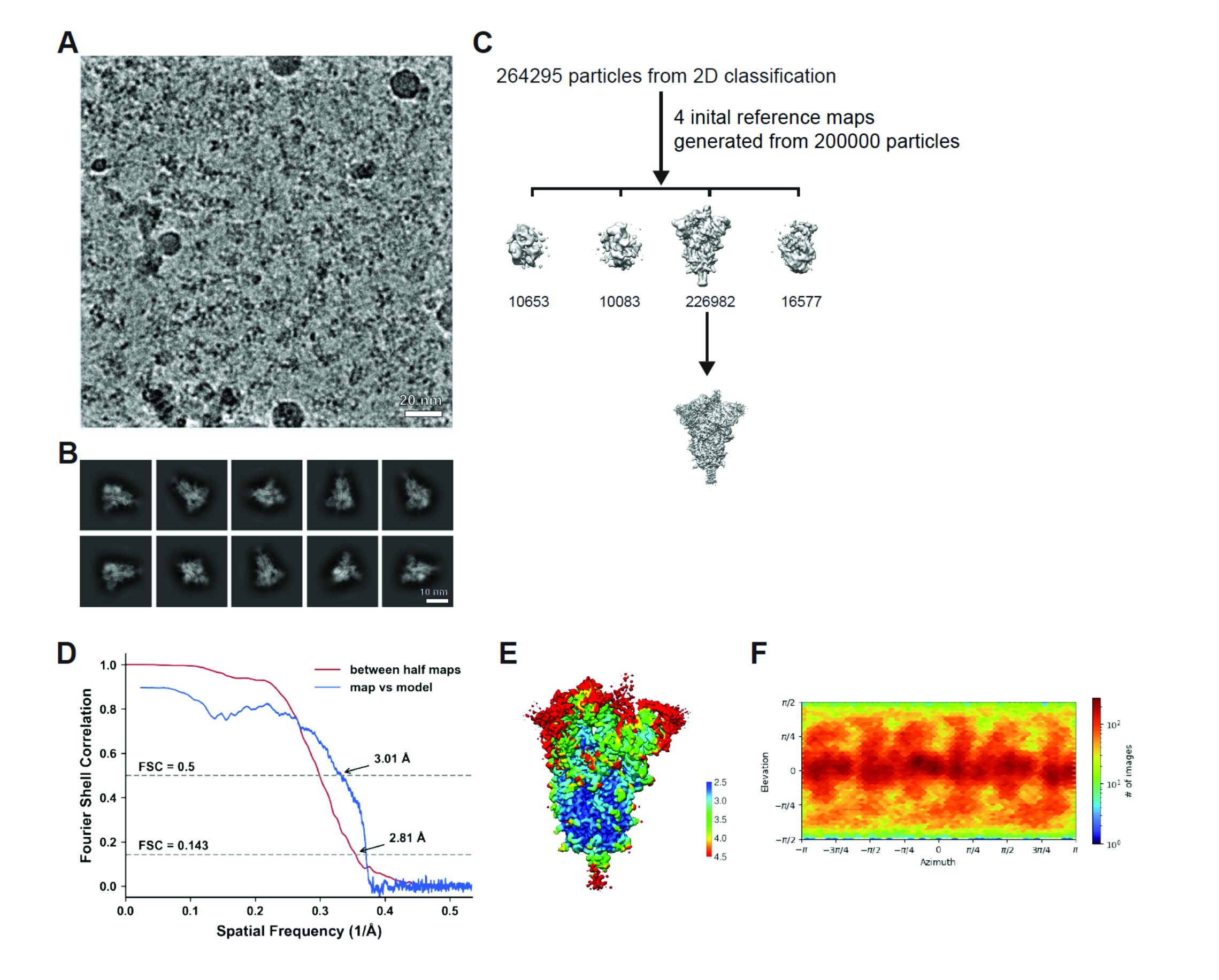

Supplement: S2 Fig — (A) Representative micrograph. (B) Representative 2D class averages. (C) Cryo-EM data processing workflow. (D) Fourier shell correlation (FSC) between 2 half maps (red) and FSC between the refined map and model (blue). (E) Local resolution estimation. (F) Viewing direction distribution. (TIF) [file pbio.3001237.s002.tif]

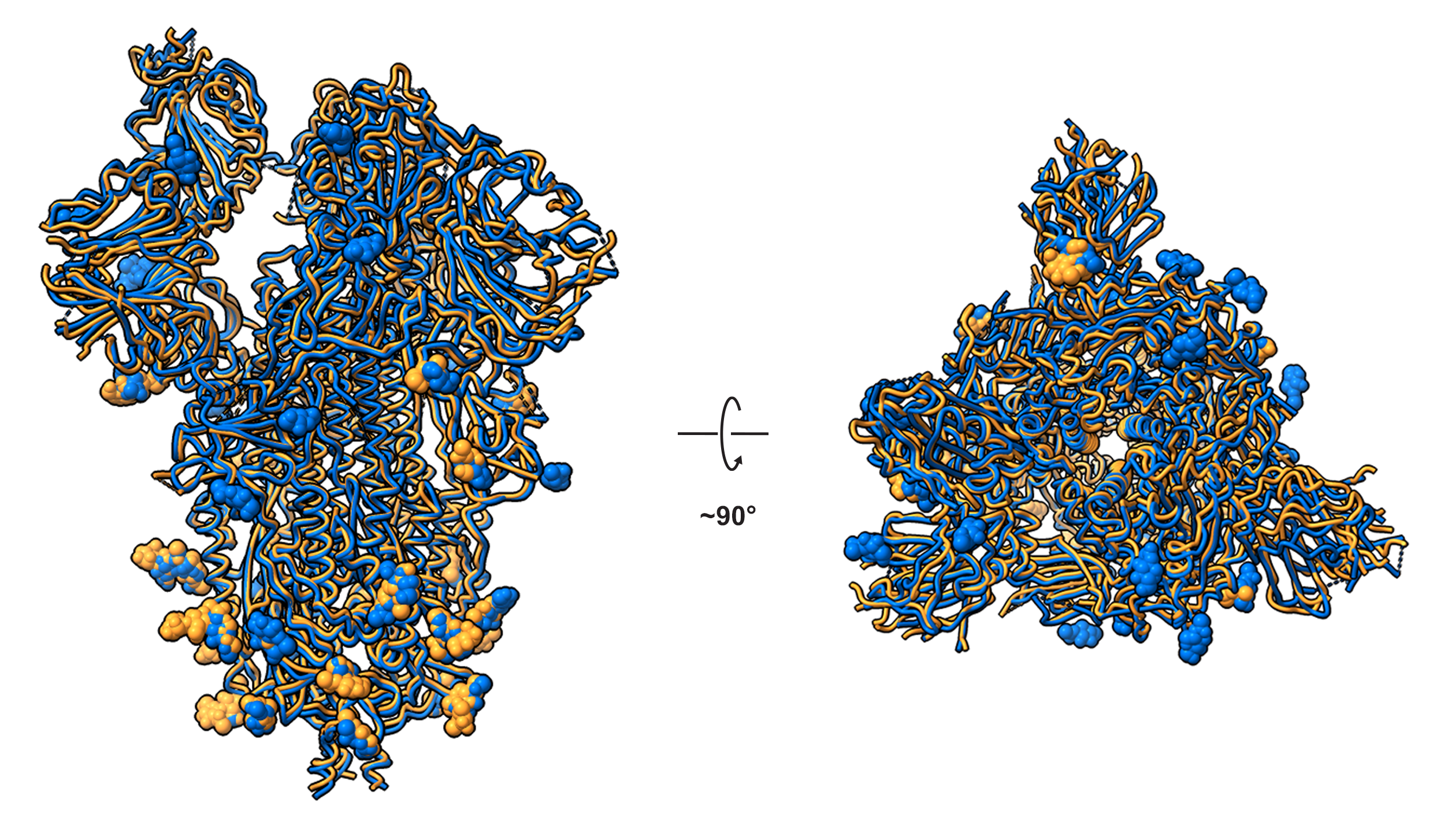

Supplement: S3 Fig — (TIF) [file pbio.3001237.s003.tif]

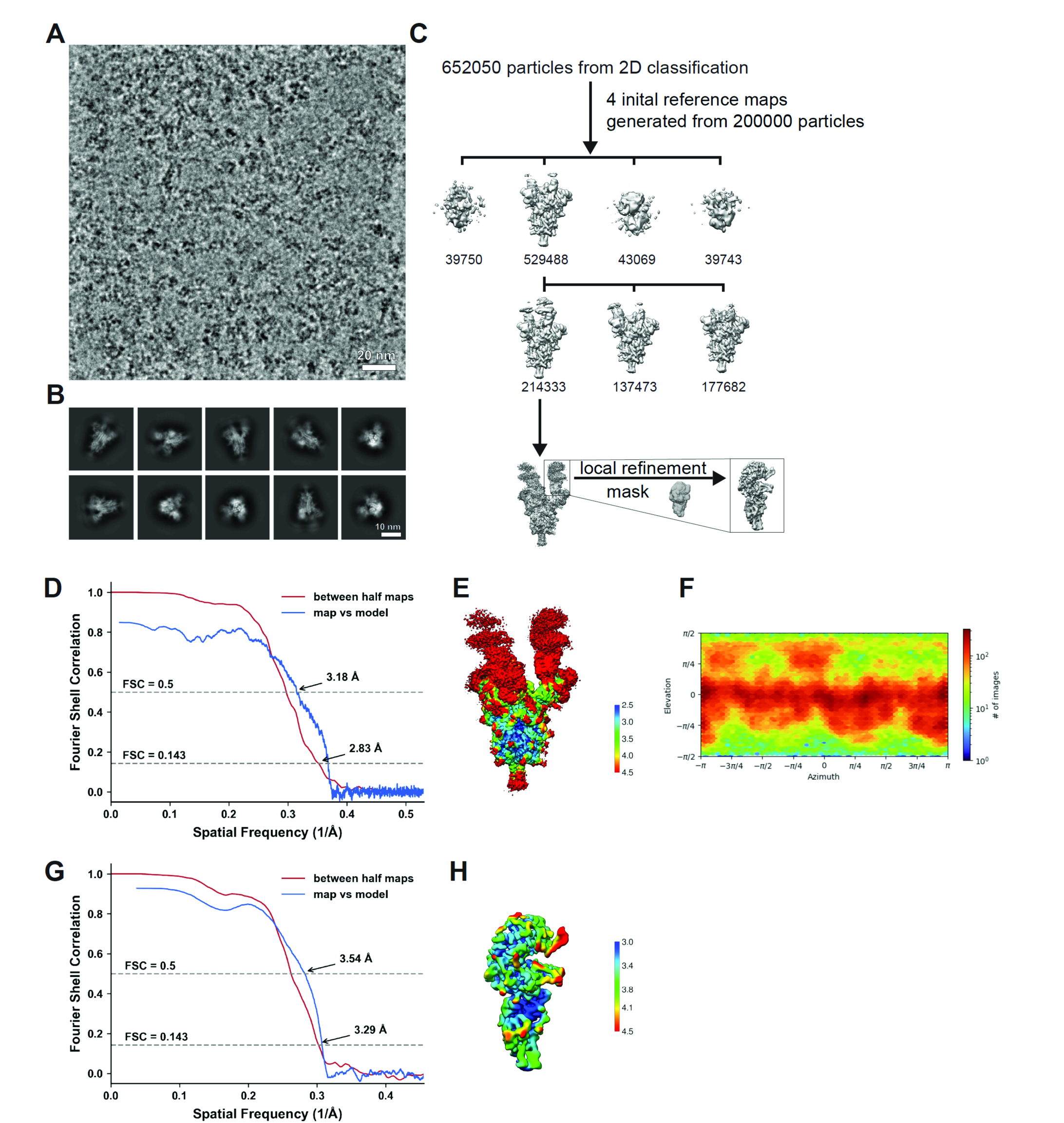

Supplement: S4 Fig — (A) Representative micrograph. (B) Representative 2D class averages. (C) Cryo-EM data processing workflow. (D) Fourier shell correlation (FSC) between 2 half maps (red) and FSC between the refined map and model (blue). (E) Local resolution estimation of the global map. (F) Viewing direction distribution. (G) FSC between 2 half maps of local refinement (red) and FSC between the locally refined map and model (blue). (H) Local resolution estimation of the locally refined map. (TIF) [file pbio.3001237.s004.tif]

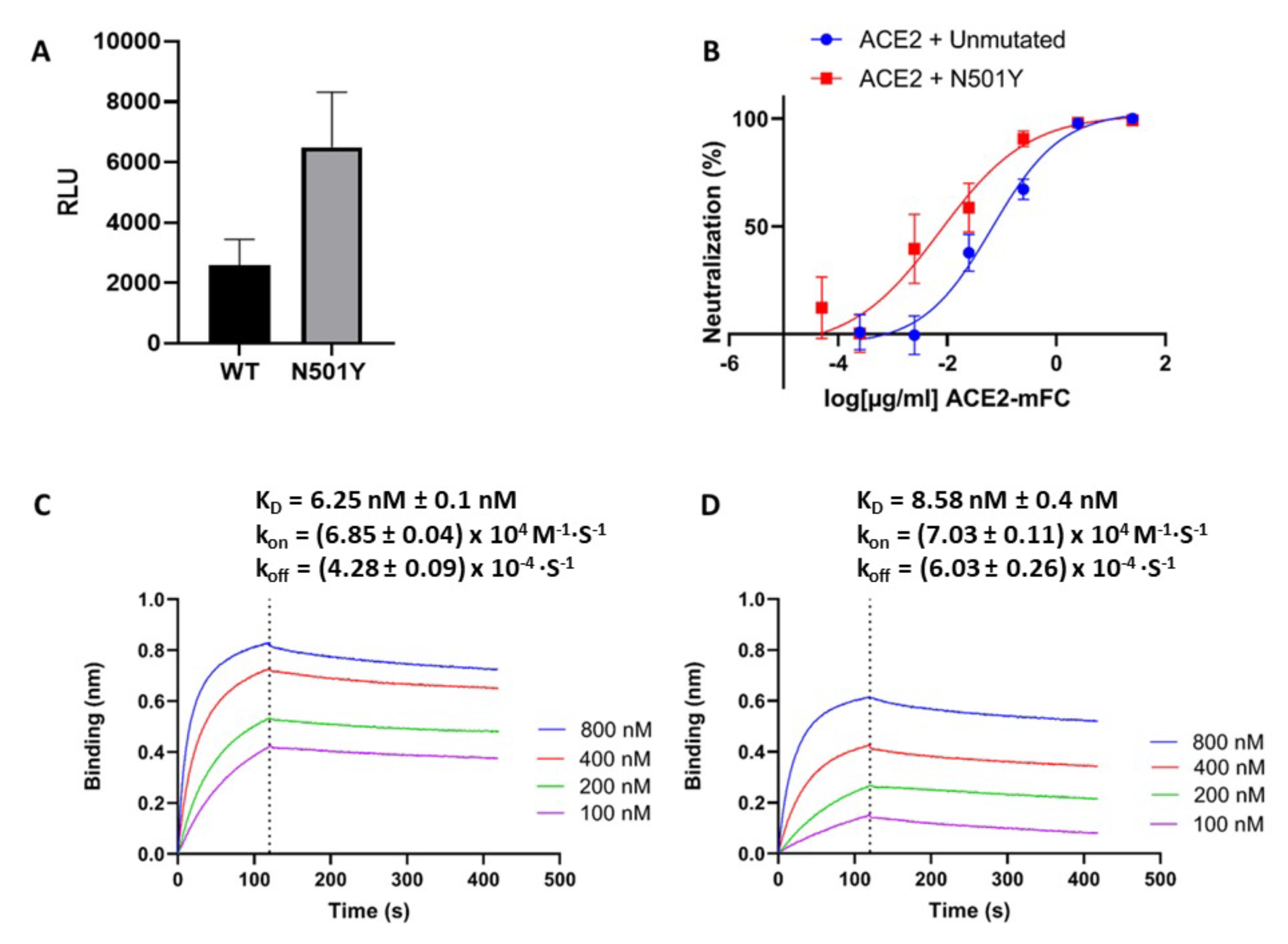

Supplement: S5 Fig — (A) Analysis of cell entry of N501Y or unmutated SARS-CoV-2 S pseudotyped viral particles. N501Y or unmutated SARS-CoV-2 S pseudotyped virus was normalized for p24 levels and incubated with HEK293T-ACE2 cells for 48 h prior to cell lysis and luciferase activity quantification. (RLU: relative luminescent units). (B) Analysis of N501Y or unmutated SARS-CoV-2 S pseudotyped virus neutralization by soluble ACE2-mFC. The IC50 of soluble ACE2-mFC neutralization is 0.066 μg/ml (95% CI 0.026–0.17 μg/ml) for unmutated pseudotyped virus, and 0.0074 μg/ml (95% CI < 0.043 μg/ml; lower bound not accurately determined) for N501Y pseudotyped virus. The IC50 for wild-type is greater than that for N501Y, as demonstrated by a 1-tailed Welch test (p = 3 × 10^−5^). (C and D) Biolayer interferometry analysis of immobilized ACE2 binding by increasing concentrations of either N501Y (A) or unmutated (B) spike ectodomain. Shown is the extent of binding as determined by shift in wavelength (nm: nanometers). Biophysical parameters (KD, kon, koff) are shown as mean ± standard deviation. (TIF) [file pbio.3001237.s005.tif]

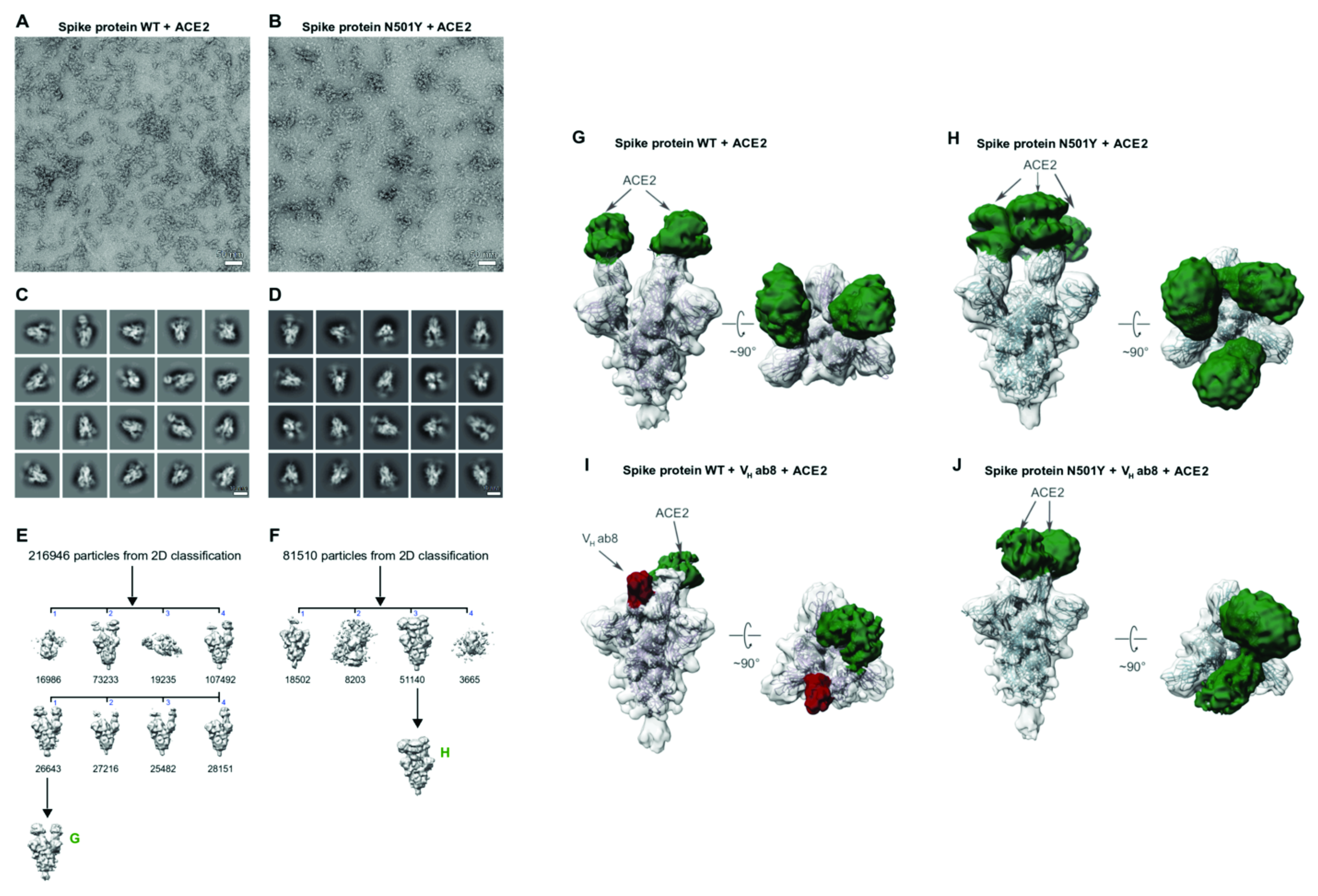

Supplement: S6 Fig — (A and B) Representative micrograph selected from the total dataset for the unmutated (A) or N501Y (B) spike ectodomains in complex with ACE2. The concentrations of spike proteins and soluble ACE2 are the same for both unmutated and N501Y preparations. (C and D) 2D class averages corresponding to (C) the unmutated dataset (1,355 images) and (D) the N501Y dataset (1,125 images), covering the same range of stain thickness. (E and F) Processing workflow. (E) For unmutated spikes, 3D classification reveals an occupancy of 2 or fewer RBDs bound for the 2 most populated initial classes (50% and 34% of all particles from 2D classification). (F) For N501Y spikes, the most populated initial class (63%) has 3 RBDs bound. (G and H) Final refinement of (G) unmutated spikes and (H) N501Y spikes. The density corresponding to bound soluble ACE2 is colored in green. The higher occupancy of ACE2 for N501Y spikes reflects a shift in the equilibrium stoichiometry, consistent with the higher affinity of N501Y spikes for ACE2. (I and J) Competition experiments. Spike ectodomains were first incubated with the VH ab8 antibody fragment, then with soluble ACE2. The density corresponding to bound VH ab8 is colored in red. The VH ab8 antibody fragment competes with ACE2 binding, as demonstrated by the reduced ACE2 occupancy in both (I) the unmutated spike (1 RBD bound) and (J) the N501Y spike (2 RBDs bound). (TIF) [file pbio.3001237.s006.tif]

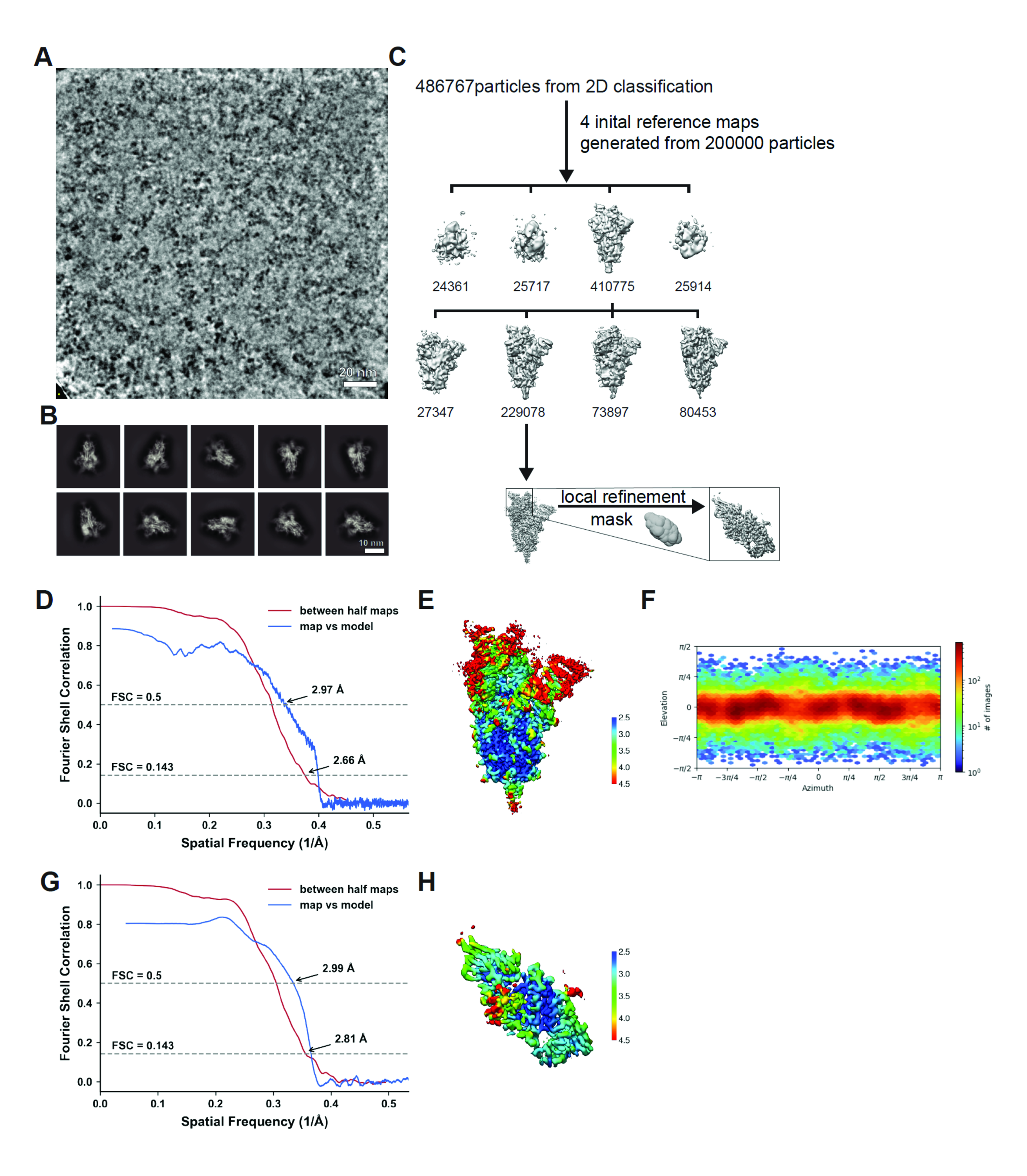

Supplement: S7 Fig — (A) Representative micrograph. (B) Representative 2D class averages. (C) Cryo-EM data processing workflow. (D) Fourier shell correlation (FSC) between 2 half maps (red) and FSC between the refined map and model (blue). (E) Local resolution estimation of the global map. (F) Viewing direction distribution. (G) FSC between 2 half maps of local refinement (red) and FSC between the locally refined map and model (blue). (H) Local resolution estimation of the locally refined map. (TIF) [file pbio.3001237.s007.tif]

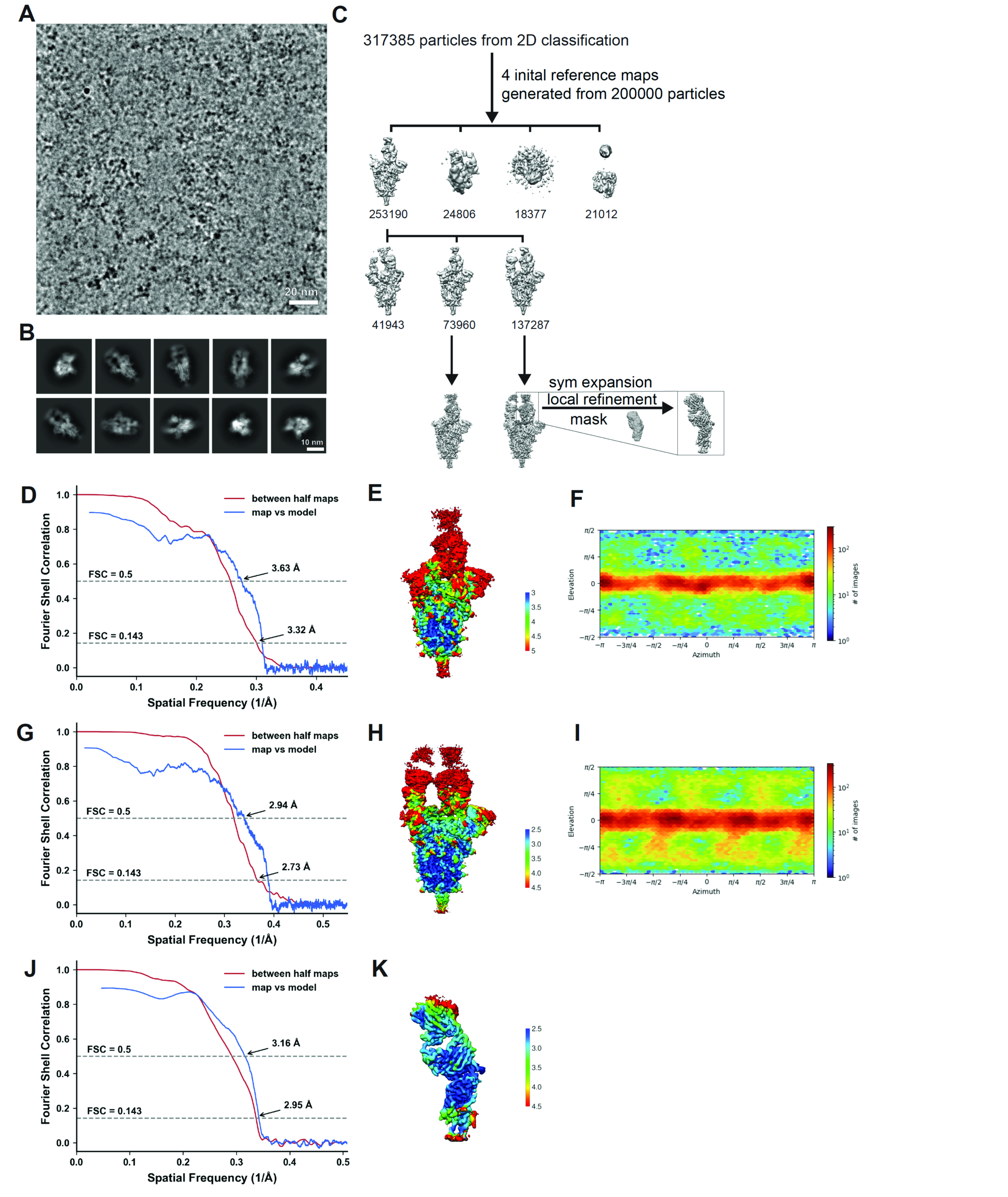

Supplement: S8 Fig — (A) Representative micrograph. (B) Representative 2D class averages. (C) Cryo-EM data processing workflow. (D–I) Fourier shell correlation (FSC) between 2 half maps (red) and between the refined map and model (blue), with local resolution estimation and viewing direction distribution, for class 1 (D–F) and class 2 (G–I). (J) FSC between 2 half maps of local refinement (red) and between the locally refined map and the model (blue). (K) Local resolution estimation of the locally refined map. (TIF) [file pbio.3001237.s008.tif]
