# Supplementary material for: Cryo-electron microscopy structures of the N501Y SARS-CoV-2 spike protein in complex with ACE2 and 2 potent neutralizing antibodies
Source: PLoS Biol. 2021 Apr 29;19(4):e3001237. doi: 10.1371/journal.pbio.3001237 (PMC8112707; doi:10.1371/journal.pbio.3001237)
Supplement: S1 Table — (DOCX) [file pbio.3001237.s009.docx]

| **Assay** | **Biolayer interferometry** | | | **Pseudovirus Neutralization** | | | **ELISA** | |
| --- | --- | --- | --- | --- | --- | --- | --- | --- |
|  | *k*_on_ | *k*_off_ | K_D_ | IC_50_ ACE2 | IC_50_ IgG ab1 | IC_50_ V_H_ ab8 | EC_50_ IgG ab1 | EC_50_ V_H_ ab8 |
|  | M^-1^·s^-1^ | s^-1^ | nM | μg/mL | nM | nM | nM | nM |
| Unmutated spikes | 7.03 | 6.03 | 8.58 | 0.066 | 0.14 | 3.76 | 0.0766 | 3.00 |
| ± Std. Dev.or CI 95% | ± 0.11 | ± 0.26 | ± 0.4 | 0.026–0.17 | 0.066–0.26 | 2.59–5.66 | 0.070–0.084 | 2.4–3.9 |
| N501Y spikes | 6.85 | 4.28 | 6.25 | 0.0074 | 0.79 | 6.61 | 0.181 | 3.75 |
| ± Std. Dev.or CI 95% | ± 0.04 | ± 0.09 | ± 0.1 | *–0.043 | 0.35–2.61 | 3.6–14.9 | 0.15–0.22 | 2.8–5.0 |

* Lower bound not accurately determined
