# Supplementary material for: Cryo-electron microscopy structures of the N501Y SARS-CoV-2 spike protein in complex with ACE2 and 2 potent neutralizing antibodies
Source: PLoS Biol. 2021 Apr 29;19(4):e3001237. doi: 10.1371/journal.pbio.3001237 (PMC8112707; doi:10.1371/journal.pbio.3001237)
Supplement: S2 Table — (DOCX) [file pbio.3001237.s010.docx]

| **ACE2**  **(6VW1)** | **VH-ab8** | **Fab-ab1** | **CC12.3**  **(6XC4)** | **CB6**  **(7C01)** | **COVA2-39**  **(7JMP)** | **BD604**  **(7CHF)** | **H11-D4**  **(6YZ5)** | **H11-H4**  **(6ZBP)** |
| --- | --- | --- | --- | --- | --- | --- | --- | --- |
|  |  |  |  |  |  |  |  | Arg346 |
|  |  | Arg403 | Arg403 | Arg403 |  | Arg403 |  |  |
|  |  | Asp405 | Asp405 | Asp405 |  | Asp405 |  |  |
|  |  |  |  | Glu406 |  |  |  |  |
|  |  |  |  | Arg408 |  |  |  |  |
|  |  |  |  | Gln409 |  |  |  |  |
|  |  | Thr415 | Thr415 | Thr415 |  | Thr415 |  |  |
|  |  | Gly416 | Gly416 |  |  | Gly416 |  |  |
|  |  | Lys417 | Lys417 | Lys417 |  | Lys417 |  |  |
|  |  | Asp420 | Asp420 | Asp420 |  | Asp420 |  |  |
|  |  | Tyr421 | Tyr421 | Tyr421 |  | Tyr421 |  |  |
|  |  |  | Gly416 |  |  |  |  |  |
|  |  |  |  |  |  |  |  | Lys444 |
|  |  |  |  |  | Gly446 |  |  |  |
| Tyr449 | Tyr449 |  |  |  | Tyr449 |  | Tyr449 | Tyr449 |
|  |  |  |  |  |  |  | Asn450 | Asn450 |
|  |  |  |  |  |  |  | Leu452 | Leu452 |
| Tyr453 |  | Tyr453 | Tyr453 |  |  | Tyr453 |  |  |
| Leu455 | Leu455 |  | Leu455 | Leu455 |  | Leu455 | Leu455 |  |
| Phe456 | Phe456 | Phe456 | Phe456 | Phe456 | Phe456 | Phe456 | Phe456 | Phe456 |
|  |  | Arg457 | Arg457 | Arg457 |  | Arg457 |  |  |
|  |  |  | Lys458 | Lys458 |  | Lys458 |  |  |
|  |  | Asn460 | Asn460 | Asn460 |  | Asn460 |  |  |
|  |  |  |  |  |  |  | Thr470 |  |
|  |  | Tyr473 | Tyr473 | Tyr473 |  | Tyr473 |  |  |
|  |  | Gln474 |  |  |  |  |  |  |
| Ala475 |  | Ala475 | Ala475 | Ala475 |  | Ala475 |  |  |
| Gly476 |  | Gly476 | Gly476 | Gly476 |  | Gly476 |  |  |
|  |  | Ser477 |  | Ser477 |  | Ser477 |  |  |
|  |  | Thr478 |  |  |  |  |  |  |
|  |  |  |  |  |  |  | Gly482 | Gly482 |
|  |  |  |  |  | Val483 |  | Val483 | Val483 |
|  | Glu484 |  |  |  | Glu484 |  | Glu484 | Glu484 |
|  | Gly485 |  |  |  | Gly485 |  |  |  |
| Phe486 | Phe486 | Phe486 | Phe486 | Phe486 | Phe486 | Phe486 |  |  |
| Asn487 | Asn487 | Asn487 | Asn487 | Asn487 | Asn487 | Asn487 |  |  |
|  | Cys488 |  |  |  |  |  |  |  |
| Tyr489 | Tyr489 | Tyr489 | Tyr489 | Tyr489 | Tyr489 | Tyr489 | Tyr489 | Tyr489 |
|  | Phe490 |  |  |  |  |  | Phe490 | Phe490 |
|  | Leu492 |  |  |  |  |  | Leu492 | Leu492 |
| Gln493 |  | Gln493 |  | Gln493 | Gln493 | Gln493 | Gln493 | Gln493 |
|  | Ser494 |  |  |  |  |  | Ser494 | Ser494 |
|  |  |  |  | Tyr495 |  |  |  |  |
| Gly496 |  |  |  |  |  | Gly496 |  |  |
| Gln498 | Gln498 | Gln498 |  |  |  | Gln498 |  |  |
| Thr500 |  |  |  |  |  | Thr500 |  |  |
| Asn501 |  | Asn501 | Asn501 |  |  | Asn501 |  |  |
| Gly502 |  |  |  |  |  | Gly502 |  |  |
|  |  |  |  |  |  | Val503 |  |  |
| Tyr505 | Tyr505 | Tyr505 | Tyr505 | Tyr505 |  | Tyr505 |  |  |
| (*10*) | This paper | This paper | (*32*) | (*42*) | (*43*) | (*44*) | (*45*) | (*45*) |

*Epitope was mapped using inputs from PDBsum(*46*).
