# Supplementary material for: Cryo-electron microscopy structures of the N501Y SARS-CoV-2 spike protein in complex with ACE2 and 2 potent neutralizing antibodies
Source: PLoS Biol. 2021 Apr 29;19(4):e3001237. doi: 10.1371/journal.pbio.3001237 (PMC8112707; doi:10.1371/journal.pbio.3001237)
Supplement: S1 Raw Images — (PDF) [file pbio.3001237.s011.pdf]

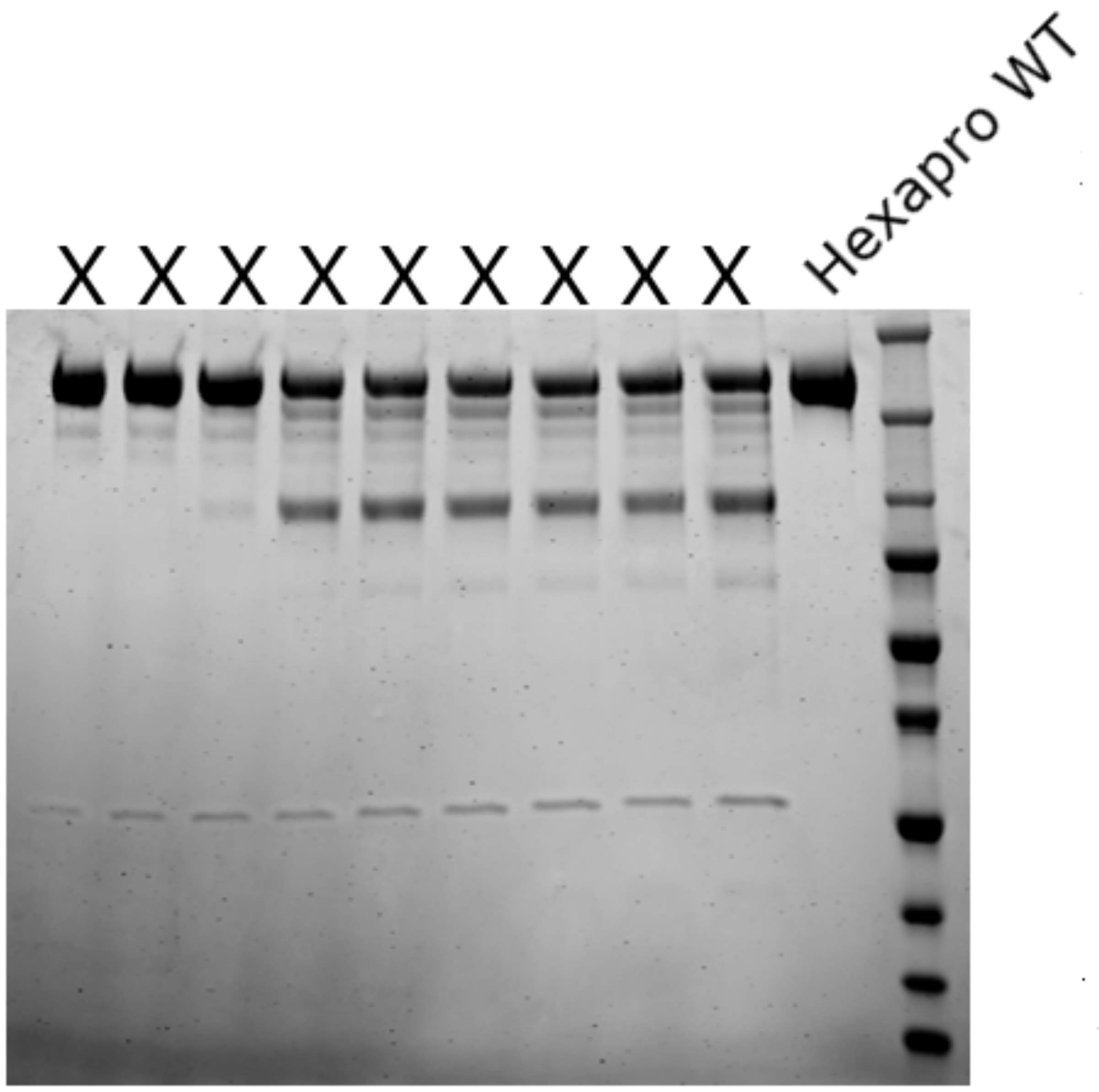

SDS-PAGE, Coomassie Stained

Related to Figure S1 B

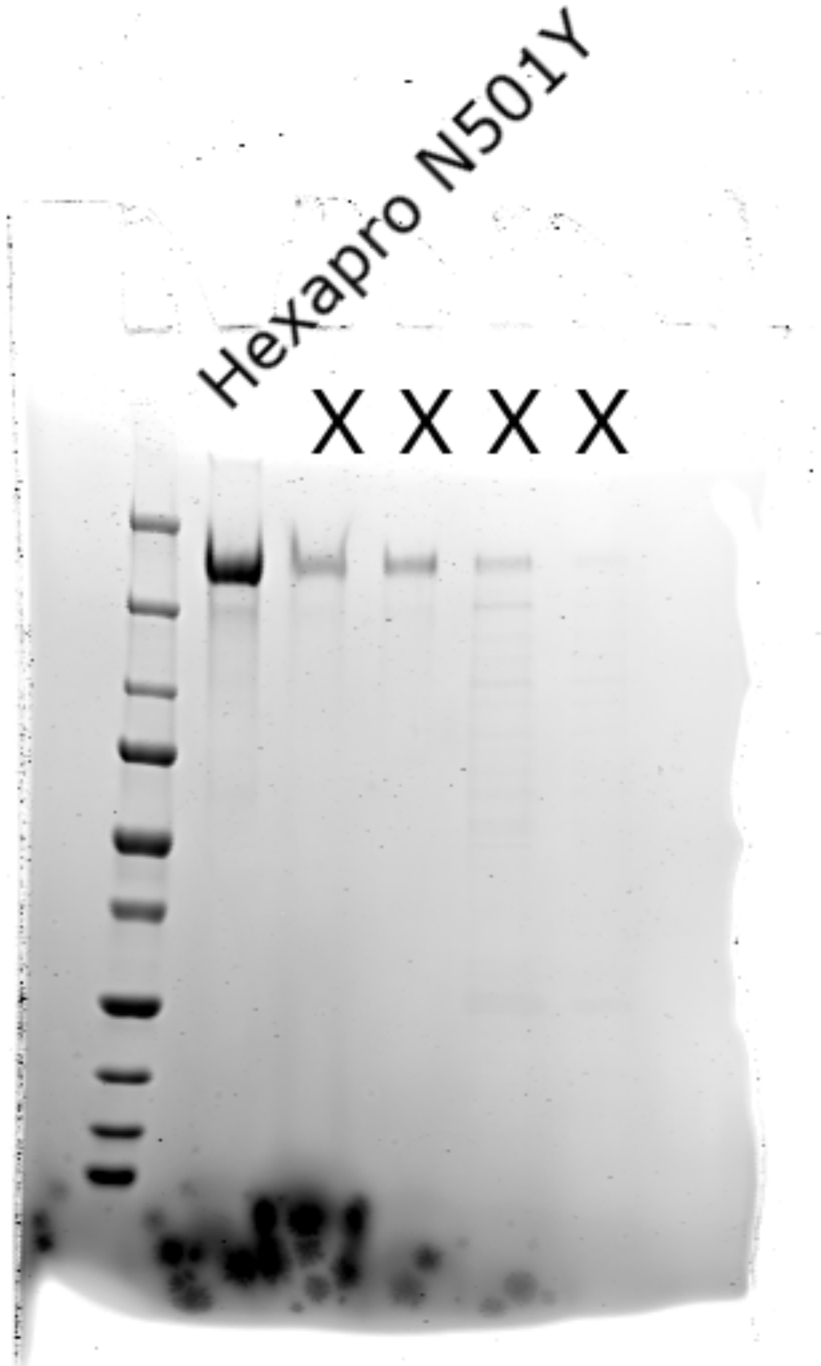

SDS-PAGE, Coomassie Stained

Related to Figure S1 B
